# Pinostrobin Suppresses the α-Melanocyte-Stimulating Hormone-Induced Melanogenic Signaling Pathway

**DOI:** 10.3390/ijms24010821

**Published:** 2023-01-03

**Authors:** Athapaththu Mudiyanselage Gihan Kavinda Athapaththu, Sobarathne Senel Sanjaya, Kyoung Tae Lee, Wisurumuni Arachchilage Hasitha Maduranga Karunarathne, Yung Hyun Choi, Sung-Pyo Hur, Gi-Young Kim

**Affiliations:** 1 Department of Marine Life Science, Jeju National University, Jeju 63243, Republic of Korea; 2 Forest Bioresources Department, Forest Microbiology Division, National Institute of Forest Science, Suwon 16631, Republic of Korea; 3 Department of Biosystems Technology, Faculty of Technology, University of Ruhuna, Matara 81000, Sri Lanka; 4 Department of Biochemistry, College of Korean Medicine, Dong-Eui University, Busan 47227, Republic of Korea

**Keywords:** pinostrobin, melanogenesis, tyrosinase, α-MSH

## Abstract

Pinostrobin is a dietary flavonoid found in several plants that possesses pharmacological properties, such as anti-cancer, anti-virus, antioxidant, anti-ulcer, and anti-aromatase effects. However, it is unclear if pinostrobin exerts anti-melanogenic properties and, if so, what the underlying molecular mechanisms comprise. Therefore, we, in this study, investigated whether pinostrobin inhibits melanin biosynthesis in vitro and in vivo, as well as the potential associated mechanism. Pinostrobin reduced mushroom tyrosinase activity in vitro in a concentration-dependent manner, with an IC_50_ of 700 μM. Molecular docking simulations further revealed that pinostrobin forms a hydrogen bond, as well as other non-covalent interactions, between the C-type lectin-like fold and polyphenol oxidase chain, rather than the previously known copper-containing catalytic center. Additionally, pinostrobin significantly decreased α-melanocyte-stimulating hormone (α-MSH)-induced extracellular and intracellular melanin production, as well as tyrosinase activity, in B16F10 melanoma cells. More specifically, pinostrobin inhibited the α-MSH-induced melanin biosynthesis signaling pathway by suppressing the cAMP–CREB–MITF axis. In fact, pinostrobin also attenuated pigmentation in α-MSH-stimulated zebrafish larvae without causing cardiotoxicity. The findings suggest that pinostrobin effectively inhibits melanogenesis in vitro and in vivo via regulation of the cAMP–CREB–MITF axis.

## 1. Introduction

Once melanin is synthesized in melanocytes, it is incorporated into the melanosome, an organelle that is transported to adjacent keratinocytes, resulting in melanin distribution [1]. Melanin, and in particular, eumelanin, protects human skin from ultraviolet radiation (UVR)-induced DNA and skin damage by absorbing UVR and scavenging UVR-induced reactive oxygen species (ROS) [2]. Hence, melanin is thought to serve as the primary photoprotective pigment that suppresses UVR-induced oxidative stress and damage. However, the unusual accumulation of melanin also causes dermatological disorders, including melasma, wrinkling, senile lentigines, and skin cancers [3,4,5]. Hence, identification and characterization of anti-melanogenic compounds has attracted considerable attention [6,7].

Tyrosinase plays an important role in increasing melanin biosynthesis through hydroxylation of tyrosine into dihydroxyphenylalanine (DOPA), followed by further oxidation into dopaquinone, which is the precursor for melanin via cysteinyl-DOPA and dopachrome, respectively [8,9]. Given that tyrosinase has been recognized as a major target molecule for the inhibition of melanin biosynthesis, many antagonists have been developed and applied clinically [2]. Tyrosinase is a di-copper oxidase in which six histidine residues surround two copper ions in its catalytically active site [10]. Goldfeder et al. [11] reported that the main substrates of tyrosinase fit in the active site, whereas the presence of Zn^2+^ ions forces out the Cu^2+^ ions, effectively inhibiting the catalytic activity. In this way, many flavonoids competitively target the active site of tyrosinase, thereby inhibiting its activity [8,9]. Accordingly, competitive inhibitors targeting tyrosinase may represent an excellent strategy for inhibiting melanin biosynthesis.

UVR increases the expression of α-melanocyte-stimulating hormone (α-MSH) in keratinocytes, which binds to the melanocortin 1 receptor (MC1R) in melanocytes and promotes melanin biosynthesis [12]. Binding of α-MSH to MC1R primarily activates adenylyl cyclase (AC), which increases intracellular cyclic 3′,5′-cyclic adenosine monophosphate (cAMP) levels and consequently stimulates protein kinase A (PKA) [13]. Subsequently, cAMP-responsive element-binding protein (CREB) is phosphorylated, which, together with CBP/p300, enhances the expression of microphthalmia-related transcription factor (MITF), a main regulator of tyrosinase expression [14]. Therefore, targeting the α-MSH-mediated signaling pathway may inhibit melanin biosynthesis by suppressing tyrosinase expression.

**Figure 1:**
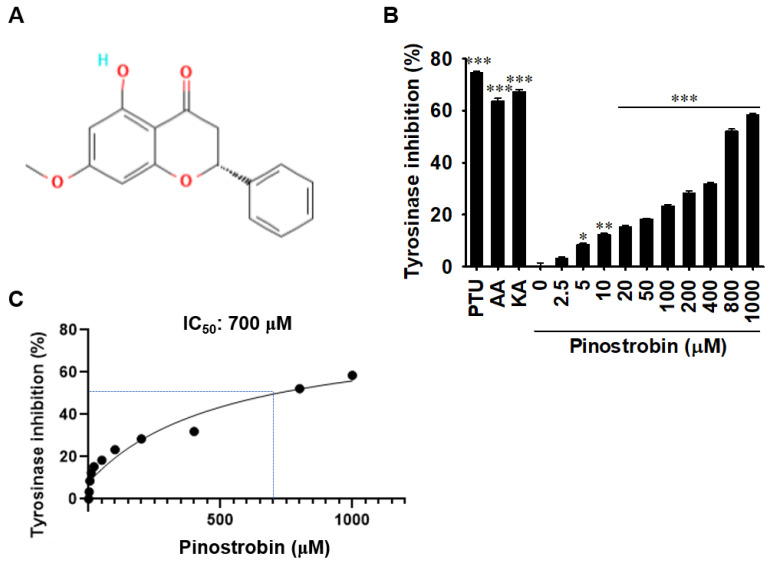
Pinostrobin inhibits in vitro mushroom tyrosinase activity. (**A**) Chemical structure of pinostrobin. (**B**) In vitro mushroom tyrosinase activity, as assessed by quantifying dopaquinone levels, following treatment with pinostrobin (0–1000 µM), phenylthiourea (PTU, 250 nM), ascorbic acid (AA, 500 µM), and kojic acid (KA, 25 µM). Data are reported as the mean ± SE. * *p* < 0.05, ** *p* < 0.01, and *** *p* < 0.001 vs. untreated control. (**C**) Pinostrobin concentration required for 50% inhibition (IC_50_).

Pinostrobin (Figure 1A) is a natural flavonoid found in various plants, such as the leaves of *Cajanus cajan* (L.) Millsp and the rhizomes of *Boesenbergia rotunda* (L.). Pinostrobin possesses a broad spectrum of pharmacological activities, including anti-cancer [15,16], antioxidant [17], anti-inflammatory [18,19], and anti-virus properties [20]. In fact, El-Nashar et al. [21] recently reported that pinostrobin, isolated from Egyptian propolis, effectively reduces in vitro mushroom tyrosinase activity. However, there is currently a dearth of data regarding the anti-melanogenic effects of pinostrobin. Therefore, in this study, we investigated whether pinostrobin downregulates melanogenesis in B16F10 melanoma cells and zebrafish larvae by inhibiting the melanogenic signaling.

## 2. Results

### 2.1. Pinostrobin Inhibits In Vitro Mushroom Tyrosinase Activity

As tyrosinase is a rate-limiting enzyme in melanogenesis [8,9], we investigated whether pinostrobin negatively regulates mushroom tyrosinase activity in vitro. As expected, the tyrosinase inhibitors phenylthiourea (PTU), ascorbic acid (AA), and kojic acid (KA) significantly inhibited mushroom tyrosinase activity by 74.7% ± 0.6%, 63.8% ± 1.0%, and 67.4% ± 0.6%, respectively (Figure 1B). Meanwhile, as the concentration of pinostrobin gradually increased, in vitro mushroom tyrosinase activity was inhibited, and 1000 μM pinostrobin exhibited the strongest inhibitory effect (58% ± 0.6%). In addition, the concentration required for 50% inhibition (IC_50_) was confirmed to be approximately 700 μM (Figure 1C). Collectively, these data suggest that pinostrobin directly inhibits tyrosinase activity in vitro at high concentrations.

### 2.2. Pinostrobin Non-Competitively Binds to Tyrosinase

**Figure 2:**
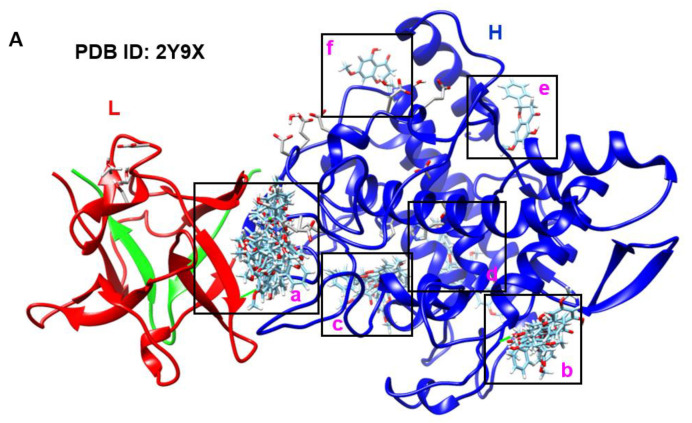

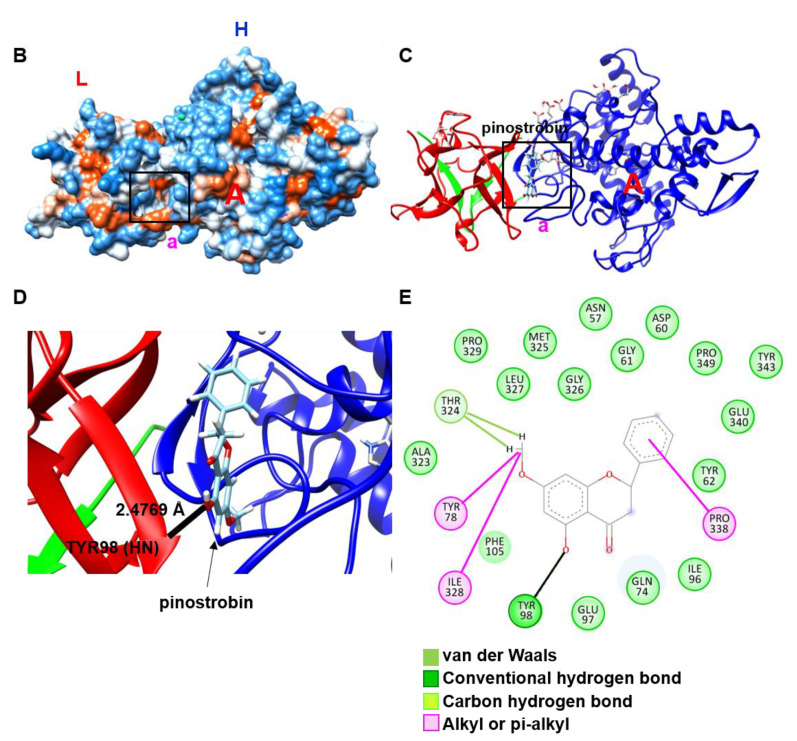
Pinostrobin non-competitively binds to mushroom tyrosinase (PDB ID: 2Y9X). (**A**) A total of 36 binding clusters of pinostrobin to mushroom tyrosinase were obtained from SwissDock. a, binding site for clusters 0, 1, 4, 8, 13, 17, 20, 21, 22, 23, 24, 27, 28, 30, and 34; b, binding site for clusters 2, 6, 9, 14, 18, 19, 31, and 33; c, binding site for clusters 7, 11, 25, 26, 32; d, binding site for clusters 12, 15, and 29; e, binding site for clusters 5 and 16; f, binding site for cluster 10. L, light chain (lectin-like fold protein); H, heavy chain (polyphenol oxidase). (**B**) The 3D conformation and (**C**) ribbon model how molecular docking site of pinostrobin (pink ‘a’) with mushroom tyrosinase. Red ‘A’ represents an active site of mushroom tyrosinase. (**D**) Enlarged binding site of pinostrobin with mushroom tyrosinase. A black line shows a hydrogen bond between pinostrobin and mushroom tyrosinase. (**E**) The 2D interaction poses of pinostrobin with mushroom tyrosinase.

Whether pinostrobin inhibits in vitro mushroom tyrosinase activity by competing with its substrate was analyzed using a protein–ligand docking simulation. Using SwissDock, 34 clusters in which pinostrobin binds to mushroom tyrosinase were identified (Figure 2A). The major binding site was identified (Figure 2A ‘a’), to which approximately 50% of clusters (0, 1, 3, 4, 8, 13, 17, 20, 21, 22, 23, 24, 27, 28, 30, and 34) were bound (Supplementary Table S1). Meanwhile, clusters 2, 6, 9, 14, 18, 19, 31, and 33 were bound to alternative binding site (Figure 2A ‘b’), where the second highest binding force was observed. Additionally, four minor pinostrobin-binding sites were identified (Figure 2A ‘c–f’). The 3D conformation (Figure 2B) and ribbon structure (Figure 2C) also showed the major pinostrobin binding site and the active site of tyrosinase containing Cu^2+^ ions. In particular, pinostrobin formed a hydrogen bond with TYR98 (HN) in the light chain (L, lectin-like fold protein) at a distance of 2.4769 Å (Figure 2D). In addition to the conventional hydrogen bond with TYR98, the 2D interaction diagram showed the formation of carbon hydrogen bonds (THR324), alkyl or π-alkyl bonds (TYR78, ILE324, and PRO338), and many van der Waals interactions with the surrounding amino acids (Figure 2E). These results indicate that pinostrobin does not compete with substrates at the active site of tyrosinase but rather primarily binds to heavy (H, polyphenol oxidase) and light chains.

### 2.3. Pinostrobin Concentrations above 100 μM Are Weakly Cytotoxic

**Figure 3:**
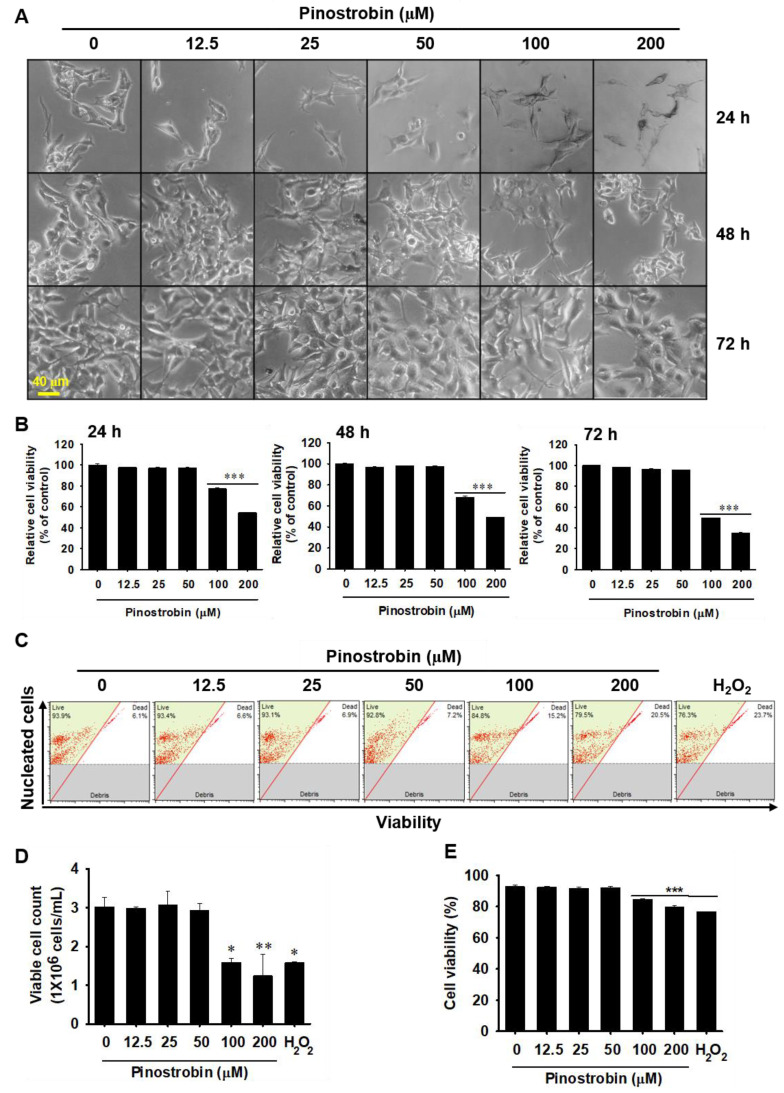
Pinostrobin at concentrations below 50 μM is not toxic to B16F10 melanoma cells. B16F10 cells were treated with pinostrobin (0–200 µM) for 72 h. (**A**) Microscopic images captured every 24 h. Scale bar = 40 μm. (**B**) Relative cell viability presented relative to the values of the untreated cells. (**C**) Treatment of cells with pinostrobin for 72 h. The cells were stained with a Muse Cell Count and Viability Kit. (**D**) Viable cell counts; (**E**) cell viability. Data are reported as mean ± SE. * *p* < 0.05, ** *p* < 0.01, and *** *p* < 0.001 vs. untreated cells.

To investigate whether pinostrobin is cytotoxic, B16F10 melanoma cells were treated with pinostrobin (0–1000 µM) for 72 h, and cytotoxicity was evaluated based on morphological changes and MTT assay. Observation under a phase-contrast microscope showed that pinostrobin treatment did not induce any morphological changes in the cells (Figure 3A). However, the MTT assay showed that treatment with pinostrobin at concentrations ≥100 μM markedly decreased the relative cell viability after 24 h (77.3% ± 1.0% and 54.3% ± 0.5% at 100 and 200 μM, respectively); the inhibitory effect became stronger after 48 h (68.3% ± 1.1% and 49.2% ± 0.2% at 100 and 200 μM, respectively) and 72 h (39.6% ± 0.1% and 30.6% ± 0.2% at 100 and 200 μM, respectively; Figure 2B).

To confirm whether pinostrobin influences cell death, flow cytometric analysis was performed (Figure 3C). Consistent with the MTT assay results, 72 h after pinostrobin treatment, when compared to untreated cells ((3.0 ± 0.2) × 10^6^ cells/mL), 100 μM and 200 μM pinostrobin significantly reduced viable cell counts ((1.6 ± 0.1) × 10^6^ cells/mL and (1.2 ± 0.6) × 10^6^ cells/mL, respectively; Figure 3D). Moreover, the viable cell population slightly decreased to 85.8% ± 0.2% and 80.9% ± 2.4% at 100 and 200 μM pinostrobin, respectively, compared to that in untreated cells (92.8% ± 0.2%; Figure 3E). H_2_O_2_ treatment for 24 h also significantly decreased viable cell counts (1.6 ± 0.6) × 10^6^ cells/mL and the viable cell population (76.7% ± 0.8%). However, pinostrobin at concentrations ≤50 μM had no effect on cell growth. These data indicate that pinostrobin at concentrations below 50 μM has no direct cytotoxic effects.

### 2.4. Pinostrobin Decreases Melanin Production and Intracellular Tyrosinase Activity

**Figure 4:**
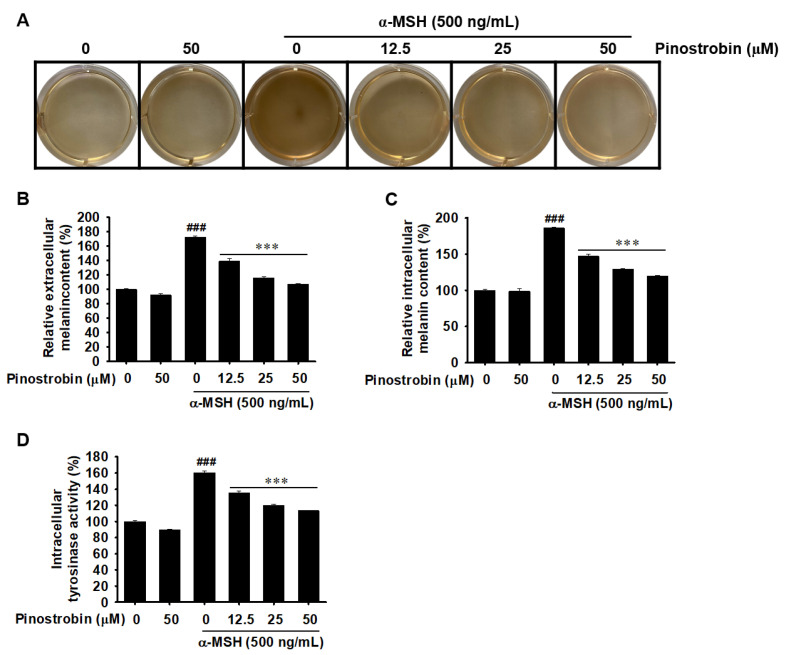
Pinostrobin decreases α-melanocyte-stimulating hormone (α-MSH)-stimulated melanin production and intracellular tyrosinase activity. B16F10 melanoma cells were stimulated with 500 ng/mL α-MSH for 24 h followed by treatment with pinostrobin (0–50 µM) for 48 h. (**A**) The color change of each well. (**B**) Extracellular melanin content following stimulation with or without α-MSH and treatment with pinostrobin. (**C**) Intracellular melanin content following stimulation with or without α-MSH and treatment with pinostrobin. (**D**) Intracellular tyrosinase activity following treatment with B16F10 cells with pinostrobin and stimulation with or without α-MSH. The data are represented as mean ± SE. ^###^ *p* < 0.001 vs. untreated cells; *** *p* < 0.001 vs. α-MSH-treated cells.

To investigate the anti-melanogenic effect of pinostrobin, α-MSH-stimulated B16F10 melanoma cells were treated with various concentrations of pinostrobin (0–50 µM) for 48 h, and the melanin content was measured in the extracellular and intracellular compartments. The degree of indirect melanin biosynthesis was assessed based on the change in media color during cell culture; a dark brown color indicates the presence of melanin. The results show a clear increase in melanin following treatment with α-MSH, which gradually decreased upon pinostrobin treatment (Figure 4A). Additionally, α-MSH significantly increased the extracellular and intracellular melanin contents to approximately 172% (Figure 4B) and 186% (Figure 4C), respectively. Meanwhile, pinostrobin downregulated α-MSH-induced extracellular and intracellular melanin content in a concentration-dependent manner. Pinostrobin (50 μM) also decreased α-MSH-induced intracellular tyrosinase activity from approximately 160% to 113% (Figure 4D). In contrast, the group treated with only 50 μM pinostrobin, without α-MSH pretreatment, exhibited melanin content and tyrosinase activity similar to those of untreated cells. These data indicate that pinostrobin inhibits extracellular and intracellular melanin production, as well as tyrosinase activity.

### 2.5. Pinostrobin Inhibits cAMP, p-CREB, MITF, and Tyrosinase Expression in α-MSH-Stimulated B16F10 Melanoma Cells

**Figure 5:**
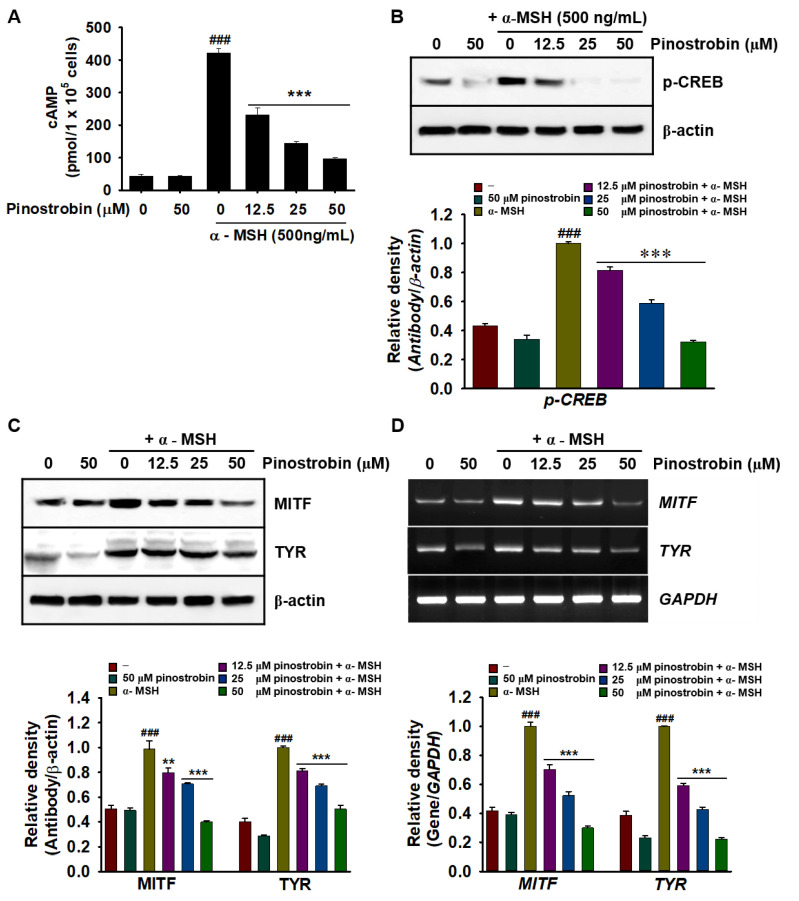
Pinostrobin inhibits α-melanocyte-stimulating hormone (α-MSH)-induced melanogenic signaling pathway. (**A**) Intracellular cAMP levels of B16F10 melanoma cells following pretreatment with IBMX for 10 min and treatment with pinostrobin in the presence or absence of **α**-MSH. (**B**,**C**) Protein abundance of phospho-cAMP-responsive element-binding protein (p-CREB), microphthalmia-related transcription factor (MITF), and tyrosinase (TYR) in B16F10 melanoma cells exposed to α-MSH and subsequently treated with pinostrobin. (**D**) Expression of *MITF* and *TYR* in B16F10 melanoma cells exposed to α-MSH and subsequently treated with pinostrobin. The data are represented as mean ± SE. ^###^ *p* < 0.001 vs. untreated cells; ** *p* < 0.01 and *** *p* < 0.001 vs. α-MSH-stimulated cells.

To elucidate the mechanism by which pinostrobin inhibits the α-MSH-induced melanogenic signaling pathway, we measured cAMP levels induced by the binding of α-MSH to MC1R. As shown in Figure 5A, pinostrobin significantly downregulated the increase in cAMP levels induced by α-MSH from 421.7 ± 14.3 pg/mL to 231.0 ± 21.7 pg/mL, 144.4 ± 5.1 pg/mL, and 96.9 ± 3.4 pg/mL at 12.5, 25, and 50 µM, respectively. Considering that upregulation of intracellular cAMP is directly associated with CREB phosphorylation, we also evaluated the effect of pinostrobin on CREB phosphorylation. α-MSH markedly increased the expression of phosphorylated CREB (p-CREB), whereas pinostrobin decreased p-CREB expression in a concentration-dependent manner (Figure 5B). Additionally, α-MSH noticeably upregulated MITF and tyrosinase at both the translational (Figure 5C) and transcriptional (Figure 5D) levels whereas pinostrobin reduced α-MSH-induced MITF and tyrosinase expression at both levels. These results indicate that pinostrobin inhibits the melanogenic signaling pathway by suppressing the cAMP–CREB–MITF–tyrosinase axis.

### 2.6. Pinostrobin Inhibits Melanin Pigmentation in Zebrafish Larvae

**Figure 6:**
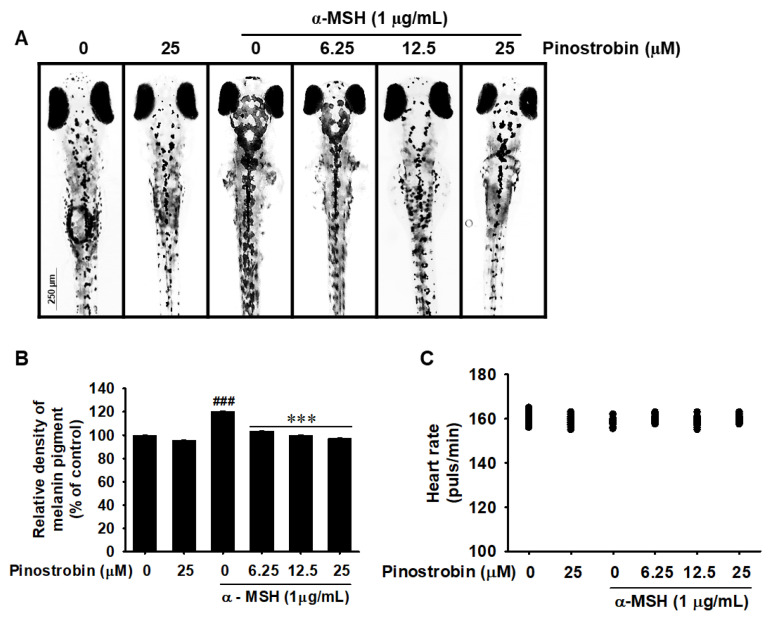
Pinostrobin inhibits melanin biosynthesis in zebrafish larvae. Zebrafish larvae at 2 dpf treated with phenylthiourea (PTU, 200 µM) and stimulated with α-melanocyte-stimulating hormone (α-MSH, 1 µg/mL). (**A**) Microscopic evaluation of zebrafish larvae pigmentation (40×). (**B**) Relative melanin pigment density. Scale bar = 250 μm. (**C**) Average heart rate of zebrafish larvae (*n =* 20). Data are reported as the mean ± SE. ^###^ *p* < 0.001 vs. untreated zebrafish larvae; *** *p* < 0.001 vs. α-MSH-stimulated zebrafish larvae.

To further evaluate the anti-melanogenic activity of pinostrobin, we treated α-MSH-stimulated zebrafish larvae with pinostrobin and quantified subsequent melanogenesis. As expected, pinostrobin markedly decreased α-MSH-induced melanin pigmentation in zebrafish larvae in a concentration-dependent manner (Figure 6A). Pinostrobin at 25 µM inhibited α-MSH-induced melanin pigmentation in untreated zebrafish larvae (Figure 6B). To determine whether pinostrobin exerted cardiotoxicity in zebrafish larvae, we monitored the heart rate and found that zebrafish larvae treated with pinostrobin did not show any apparent difference compared to that in the untreated larvae (Figure 6C). Furthermore, neither morphological malformations nor mortality of the larvae were observed following treatment with pinostrobin for 48 h. These results suggest that pinostrobin is also a potent inhibitor of melanogenesis in vivo.

## 3. Discussion

UV exposure promotes α-MSH expression in keratinocytes, which stimulates the melanogenic signaling pathway by binding to MC1R in melanocytes [1,22]. The melanin produced by melanocytes spreads to the epidermis via keratinocytes and protects cells against UV-induced ROS stress and apoptosis [2,23]. However, abnormal hyperpigmentation creates skin darkness, such as spots or patches, by increasing melanin production when skin cells are severely damaged [24]. In addition to skin injury, accumulated melanin also induces acquired hyperpigmentation disorders, including metabolic, endocrine, and nutritional disorders [25]. Therefore, many drugs that can effectively inhibit tyrosinase activity with few side effects have been developed and are currently being used in clinical practice [26]. In this respect, many flavonoids that target tyrosinase have been evaluated [21]. Although several previous studies have demonstrated that pinostrobin inhibits tyrosinase activity in vitro [21,27], the exact molecular mechanism has not been studied. In this study, we determined that pinostrobin inhibits α-MSH-induced melanogenic activity. Contrary to our findings, Yoon et al. recently confirmed that pinostrobin stimulates spontaneous intracellular melanin content and tyrosinase activity accompanied by high expression of MITF and CREB [28]. The authors also showed no cytotoxic effect in pinostrobin-treated B16F10 cells using MTT assay. In our study, high concentrations of pinostrobin significantly decreased relative cell viability using the MTT assay (precisely, it shows NADPH-dependent cellular oxidoreductase enzyme activity, not viability), as well as total cell numbers using flow cytometry; however, flow cytometry data also showed that pinostrobin slightly downregulated viable cell population, which indicates that pinostrobin disturbs or arrests the cell cycle, and does not show strong cytotoxicity, as reported previously [29]. It is unknown whether cell cycle retardation results in discrepancies regarding anti-melanogenic or melanogenic effects of pinostrobin, but future studies are needed.

Tyrosinase is a rate-limiting enzyme for melanin biosynthesis and contains copper ions at the catalytic center, which catalyze the hydroxylation and oxidation of substrates, including L-tyrosine and L-DOPA, to form dopachrome, resulting in melanin biosynthesis [30]. Therefore, many inhibitors targeting the catalytic center in tyrosinase have been developed [11,31,32]. In particular, many natural flavonoids fit into the catalytic pocket and are suitable chelators of Cu^2+^ using a flavonoid core and ionizable OH substituents. Hence, many flavonoids inhibit tyrosinase activity by competing with substrates at their catalytic site [33,34,35]. In this study, we demonstrated that pinostrobin inhibits mushroom tyrosinase activity in vitro, with an IC_50_ higher than that of flavonoids previously reported in other studies [36,37]. Molecular docking simulations confirmed the binding site of pinostrobin in tyrosinase. Unlike previously known flavonoids, pinostrobin does not fit into the active and catalytic centers, but rather binds primarily to another site between the light chain (lectin-like fold protein) and heavy chain (polyphenol oxidase). Whether this binding induces a conformational change in tyrosinase and acts as a non-competitive inhibitor is currently unknown. In addition, in order to accurately predict molecular docking between pinostrobin and tyrosinase, molecular dynamics and in silico molecular docking studies should be conducted.

Numerous cell signaling pathways are also involved in melanin biosynthesis. Among them, α-MSH-mediated upregulation of MITF is considered a key inducer of melanogenesis [38]. The binding of α-MSH to MC1R promotes cAMP formation and subsequently activates PKA-mediated CREB phosphorylation, which consequently transactivates MITF [13]. Activated MITF subsequently enhances the transcription of melanogenesis-promoting proteins such as tyrosinase [39]. Liu-Smith and Meyskens [40] previously reported that flavonoids inhibit melanin biosynthesis by suppressing the cAMP–PKA–CREB–MITF–tyrosinase signaling pathway, leading to excellent effects in the prevention and treatment of melanoma. In this study, we found that pinostrobin effectively inhibited α-MSH-mediated melanin biosynthesis, both in vitro and in vivo, by suppressing tyrosinase expression and activity. Unlike previous studies [36,41], pinostrobin effectively inhibits melanin biosynthesis in α-MSH-treated B16F10 melanoma cells and zebrafish larvae at 50 and 25 μM, respectively, which is relatively low compared with the IC_50_ (approximately 700 μM) of in vitro mushroom tyrosinase. These results suggest that pinostrobin exerts its effect predominantly via interference with the melanin biosynthesis cell signaling pathway rather than through direct inhibition of tyrosinase activity. Additionally, Pillaiyar et al. [42] reported that several soluble factors, such as wingless-related integration site, stem cell factor, and endothelin-1, stimulate MITF-mediated melanogenesis in melanocytes. Therefore, it is necessary to determine whether pinostrobin also contributes to the inhibition of other signaling pathways in addition to inhibiting the α-MSH-induced melanogenesis signaling process. Furthermore, we need to perform the more preclinical experiment to identify the physiological concentration of pinostrobin to inhibit α-MSH-induced melanogenesis.

## 4. Materials and Methods

### 4.1. Regents and Antibodies

Dulbecco’s Modified Eagle’s Medium (DMEM), fetal bovine serum (FBS), and an antibiotic mixture were purchased from WeLGENE (Gyeongsan, Gyeongsangbuk-do, Republic of Korea). PTU, AA, KA, mushroom tyrosinase, 3-(4,5-dimethylthiazol-2-yl)-2,5-diphenyltetrazolium bromide (MTT), α-MSH, and 3-isoburyl-1-methylxanthine (IBMX) were purchased from Sigma-Aldrich (St. Louis, MO, USA). Antibodies against tyrosinase (sc-20035), MITF (sc-71588), p-CREB (sc-81486), and β-actin (sc-69879) were obtained from the Santa Cruz Biotechnology (Dallas, TX, USA). Pinostrobin (purity: ≥99%) was purified from the stems of *Prunus serrulata* Lindl. F. *serrulata spontanea* (Maxim.) Chin S. Chang according to a previous method [43] and was provided from the National Institute of Forest Science (Suwon, Gyeonggi-do, Republic of Korea). All other chemicals were purchased from Sigma-Aldrich.

### 4.2. In Vitro Mushroom Tyrosinase Assay

Tyrosinase inhibition was measured using mushroom tyrosinase in a cell-free system by modifying the method of Duckworth and Coleman [44]. Briefly, the reaction mixture was prepared with 130 µL of 100 mM phosphate buffer (pH 6.8), 20 µL of pinostrobin, 30 µL of 1.5 mM L-tyrosine, and 20 µL of 210 U/mL mushroom tyrosinase and incubated for 30 min at 37 °C, and absorbance was measured at 490 nm using a microplate spectrophotometer (Thermo Fisher Scientific, Rockford, IL, USA). PTU (250 nM), AA (500 µM), and KA (25 µM) were used as positive controls. The inhibition rate (%) of mushroom tyrosinase in vitro was calculated using Equation (1):

Inhibition (%) = [A_0_ − (A_1_ − A_2_)/A_0_] − 100 (1)

where A_0_, A_1_, and A_2_ are the absorbance values of the control ((L-tyrosine + tyrosinase) − L-tyrosine), samples (L-tyrosine + samples + tyrosinase), and blank (L-tyrosine + samples), respectively. IC_50_ was calculated using GraphPad Prism 9.3 (San Diego, CA, USA).

### 4.3. Molecular Docking

The crystal structure of tyrosinase from *Agaricus bisporus* [protein database bank (PDB) ID: 2Y9X] was obtained from the RCSB PDB, and the chemical structure of pinostrobin (CID: 73201) was obtained from PubChem (https://pubchem.ncbi.nlm.nih.gov). For protein–ligand docking, simulations were performed using SwissDock (swissdockd_6tLCTN_0RI6WK5VODF69B6RFS4J, accessed on 23 December 2022) [45]. A monomer of 2Y9X (a heavy (polyphenol oxidase) and a light (lectin-like fold protein) chain) was as the full structure of 2Y9X could not be uploaded to SwissDock. Molecular docking data were visualized using the UCSD Chimera. A 2D interaction diagram was constructed using Discovery Studio Visualizer (https://www.discover.3ds.com/discovery-studio-visualizer-download).

### 4.4. Cell Culture and Cell Viability Assay

Murine B16F10 melanoma cells (ATCC, Manassas, VA, USA) were maintained in DMEM supplemented with 10% heat-inactivated FBS at 37 °C in a humidified atmosphere containing 5% CO_2_. To analyze the effect of pinostrobin on cell viability, an MTT assay was performed. Briefly, B16F10 melanoma cells were seeded in 24-well plates at a density of 1 × 10^4^ cells/mL for 16 h. The cells were then treated with the indicated concentrations of pinostrobin (0–200 μM) for 24, 48, and 72 h. After incubation, MTT was added to each well and the plates were incubated for 4 h at 37 °C. The precipitate was dissolved in dimethyl sulfoxide (DMSO), and the absorbance was measured at 540 nm using a microplate spectrophotometer (Thermo Fisher Scientific, Waltham, MA, USA). Cellular morphology was observed under a stereomicroscope (MACROTECH, Goyang, Gyeonggi-do, Republic of Korea).

### 4.5. Flow Cytometry Analysis

To estimate the total viable cell count and viable cell population, flow cytometry was performed. Briefly, B16F10 melanoma cells were plated at a density of 1 × 10^4^ cells/mL and treated with the indicated concentrations of pinostrobin (0–200 μM) for 72 h. Hydrogen peroxide (H_2_O_2_, 100 μM) was added for 24 h and used as a cell death-inducing control. Briefly, the cells were harvested and washed with ice-cold phosphate-buffered saline (PBS). The cells were then incubated with a Muse Cell Count and Viability Kit (Luminex, Austin, Texas, USA) for 5 min and analyzed using a Muse Cell Analyzer (Luminex).

### 4.6. Measurement of Extracellular and Intracellular Melanin Content

Melanin content was measured as previously described [46]. B16F10 melanoma cells were cultured at 1 × 10^4^ cell/mL in 6-well plates for 16 h and treated with α-MSH (500 ng/mL) for 24 h, followed by treatment with the indicated concentrations of pinostrobin (0–50 μM) for 48 h. Extracellular melanin content was measured using culture media at 405 nm. To measure the intracellular melanin content, the cells were washed in ice-cold PBS and dissolved in 1 N NaOH containing 10% DMSO at 100 °C for 10 min. The dissolved melanin content was measured at 405 nm.

### 4.7. Measurement of Intracellular Tyrosinase Activity

Intracellular tyrosinase activity was measured as previously described [47]. Briefly, B16F10 melanoma cells (5 × 10^4^ cells/mL) were pretreated with 500 ng/mL α-MSH for 24 h and the indicated concentrations of pinostrobin (0–50 µM) were incubated for 48 h. The cells were then lysed with PBS containing 1% Triton X-100 by freezing at −20 °C for 2 h and disrupted by thawing at room temperature. Total protein was quantified using Bio-Rad Protein Assay Reagents (Bio-Rad, Hercules, CA, USA) and an equal amount of protein was mixed with 90 µL of 5 mM L-DOPA at 37 °C for 30 min. Absorbance was measured at a wavelength of 405 nm.

### 4.8. Enzyme-Linked Immunosorbent Assay (ELISA) for cAMP

B16F10 melanoma cells (5 × 10^4^ cells/mL) were cultured in serum-free DMEM media and pretreated with 1 mM IBMX for 10 min. Pinostrobin (0–50 µM) was then added in the presence or absence of 500 ng/mL α-MSH for 15 min. Intracellular cAMP levels were measured using a colorimetric ELISA kit (Cell Biolabs Inc., San Diego, CA, USA). Finally, the absorbance was measured at 450 nm and the amount of cAMP was calculated using a cAMP standard curve.

### 4.9. Reverse Transcription-Polymerase Chain Reaction (RT-PCR)

B16F10 melanoma cells were seeded at 1 × 10^4^ cells/mL in 6-well plates and pretreated with α-MSH (500 ng/mL) 24 h before treatment with pinostrobin (0–50 μM) for 48 h. Total RNA was extracted using an easy-BLUE Total RNA Extraction Kit (iNtRON Biotechnology, Seongnam, Gyeonggi-do, Republic of Korea), according to the manufacturer’s protocol. The sequences of the sense and antisense primers were as follows: tyrosinase (*TYR*) sense 5′-GTCGTCACCCTGAAAATCCTAACT-3′ and antisense 5′-CATCGCATAAAACCTGATGGC-3′; *MITF* sense 5′-CCCGTCTCTGGAAACTTGATCG-3′ and antisense 5′-CTGTACTCTGAGCAGCAGGTC-3′; glyceraldehyde-3-phosphate dehydrogenase (*GAPDH*) sense 5′-AGGTCGGTGTGAACGGATTTG-3′ and antisense 5′-TGTAGACCATGTAGTTGAGGTCA-3′ [48]. PCR was conducted using a Veriti 96-well thermal cycler (Thermo Fisher Scientific). The PCR products were visualized using ethidium bromide.

### 4.10. Western Blot Analysis

B16F10 melanoma cells were seeded at a density of 1 × 10^4^ cell/mL in 6-well plates. The cells were then pretreated with α-MSH (500 ng/mL) for 24 h before treatment with pinostrobin (0–50 μM) for 48 h and subsequently lysed using PRO-PREP lysis buffer (iNtRON Biotechnology). The supernatant was collected, and protein concentrations were measured using Bio-Rad protein assay reagents (Bio-Rad). Equal amounts of protein (25 µg) were separated on SDS–polyacrylamide gels, transferred to nitrocellulose membranes (Schleicher & Schuell, Keene, NH, USA), and immunoblotted with specific antibodies. The bound antibodies were detected using an enhanced chemiluminescence plus kit (Thermo Fisher Scientific). Images were visualized and captured using ImageQuant LAS 500 (GE Healthcare Bio-Sciences AB, Uppsala, Sweden).

### 4.11. Maintenance of Zebrafish

AB strain zebrafish were provided by C.H. Kang (Nakdong National Institute of Biological Resources, Sangju, Gyeongsangbuk-do, Republic of Korea) and cultured at 28 °C under a 14/10 h light/dark cycle. Zebrafish were handled according to the standard guidelines of the Animal Care and Use Committee of Jeju National University (approval No. 2022-0036). Zebrafish housing and husbandry was followed by the recommendations [49]. Embryos obtained from natural spawning in embryo medium [34.8 g NaCl, 1.6 g KCl, 5.8 g CaCl_2_·2H_2_O, and 9.78 g MgCl_2_·6H_2_O in double-distilled water, pH 7.2]. The media was supplemented with 1% methylene blue at 28 °C.

### 4.12. Melanogenesis and Heart Rate in Zebrafish

One day post-fertilization (dpf) zebrafish larvae (*n =* 20) were pretreated with PTU (200 µM) for 48 h and then incubated with α-MSH (1 µg/mL) for an additional 24 h. At 4 dpf, the medium was replaced with PTU and α-MSH, and the embryos were treated with pinostrobin (0–25 μM) for 48 h (at 6 dpf). After anesthetizing zebrafish larvae with 0.02% tricane methanesulfonate, they were mounted in 2% methyl cellulose on a depression slide, and images were collected using stereomicroscopye (Tokyo, Japan). Densitometric analysis was performed using the ImageJ software (National Institutes of Health, Bethesda, MA, USA). The quantification of pigmentation data was calculated as a percentage of untreated zebrafish larvae. In a parallel experiment, heart rate was manually calculated using a stereomicroscope. The results are represented as the average heart rate per minute.

### 4.13. Statistical Analysis

All data in this study represent the mean of triplicate experiments and are expressed as mean ± standard error (SE). Statistical analysis was performed using Sigma plot 12.0 (Systat Software Inc., San Jose, CA, USA) by Student’s *t*-test and unpaired one-way analysis of variance (ANOVA) with Bonferroni’s correction.

## 5. Conclusions

**Figure 7:**
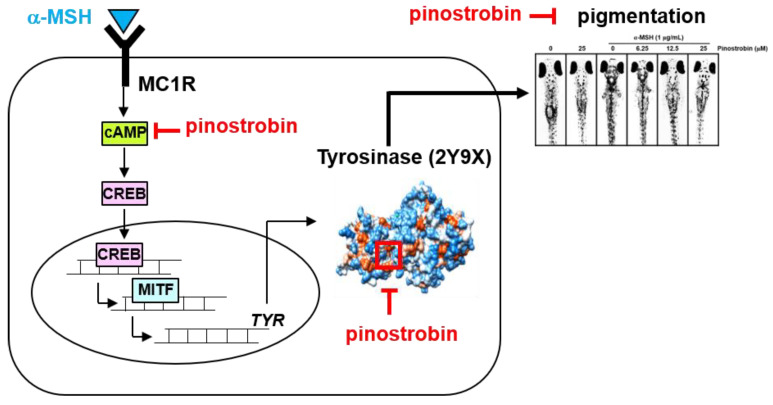
A scheme showing the molecular action of pinostrobin in inhibiting melanogenesis.

In conclusion, this study demonstrates that pinostrobin potently inhibits melanin biosynthesis in vitro and in vivo, thus eliciting a significant anti-melanogenic effect (Figure 7). This suggests that pinostrobin has the potential for novel clinical applications in the prevention and treatment of dermatological disorders, including wrinkling, melasma, and senile lentigines.

## Data Availability

All data analyzed in this study are available form the corresponding author (immunkim@jejunu.ac.kr) on reasonable request.

## Supplementary Materials

The supporting information can be downloaded at: https://www.mdpi.com/article/10.3390/ijms24010821/s1.
