# Supplementary material for: Pinostrobin Suppresses the α-Melanocyte-Stimulating Hormone-Induced Melanogenic Signaling Pathway
Source: Int J Mol Sci. 2023 Jan 3;24(1):821. doi: 10.3390/ijms24010821 (PMC9821324; doi:10.3390/ijms24010821)
Supplement: Supplementary file 1 [file ijms-24-00821-s001.zip › ijms-2068610-supplementary.pdf]

Supplementary Table S1. Docking cluster, element, fullfitneess, estimate ΔG between 2Y9X and pinostrobin using SwissDock.

Cluster	Element	FullFitness (kcal/mol)	Estimated ΔG (kcal/mol)	Cluster	Element	FullFitness (kcal/mol)	Estimated ΔG (kcal/mol)	Cluster	Element	FullFitness (kcal/mol)	Estimated ΔG (kcal/mol)
0	0	-940.18	-6.48	10	0	-936.46	-6.71	21	5	-933.61	-5.8
0	1	-940.18	-6.48	10	1	-916.21	-5.64	21	6	-933.61	-5.8
0	2	-938.88	-6.28	11	0	-936.05	-6.47	21	7	-933.58	-5.79
0	3	-938.88	-6.28	11	1	-936.03	-6.45	22	0	-933.93	-6.24
0	4	-937.53	-6.12	11	2	-935.93	-6.45	22	1	-933.86	-6.25
0	5	-937.53	-6.12	11	3	-935.93	-6.45	22	2	-933.85	-6.26
0	6	-937.53	-6.12	11	4	-933.76	-5.98	22	3	-933.81	-6.24
0	7	-937.53	-6.12	11	5	-933.62	-6	22	4	-933.58	-6.22
1	0	-940.08	-7.5	11	6	-933.41	-5.97	22	5	-929.12	-6.26
1	1	-939.94	-7.48	11	7	-933.41	-5.97	22	6	-927.83	-5.82
1	2	-939.85	-7.49	11	8	-933.27	-6.21	22	7	-927.74	-5.87
1	3	-939.43	-7.48	11	9	-932.92	-5.96	23	0	-933.9	-6.27
1	4	-939.11	-7.46	11	10	-932.76	-5.92	23	1	-933.86	-6.27
1	5	-931.88	-6.53	11	11	-932.67	-5.92	23	2	-933.84	-6.25
1	6	-931.5	-6.92	11	12	-932.65	-5.93	23	3	-931.07	-6.43
2	0	-937.93	-7.2	12	0	-935.7	-6.26	23	4	-930.61	-6.24
2	1	-933.86	-6.52	12	1	-935.67	-6.25	23	5	-930.6	-6.46
2	2	-933.8	-6.5	12	2	-935.6	-6.23	23	6	-930.55	-6.46
2	3	-933.71	-6.5	12	3	-930.39	-5.75	23	7	-930.55	-6.24
2	4	-923.01	-5.37	12	4	-929.84	-5.66	24	0	-933.76	-5.86
2	5	-922.98	-5.37	12	5	-929.53	-5.61	24	1	-933.47	-5.83
2	6	-922.97	-5.36	12	6	-929.34	-5.61	24	2	-933.47	-5.83
2	7	-921.46	-5.33	12	7	-929.2	-5.58	24	3	-933.45	-5.83

3	0	-937.83	-6.51	13	0	-935.46	-5.88	24	4	-933.45	-5.83
3	1	-936.12	-6.39	13	1	-935.46	-5.88	24	5	-933.45	-5.83
3	2	-936.12	-6.39	13	2	-935.46	-5.88	24	6	-933.45	-5.83
3	3	-936.12	-6.39	13	3	-935.46	-5.88	24	7	-933.45	-5.83
3	4	-935.67	-6.36	13	4	-935.46	-5.88	25	0	-933.43	-6.06
3	5	-935.67	-6.36	13	5	-935.45	-5.88	25	1	-933.43	-6.06
3	6	-935.67	-6.36	13	6	-935.45	-5.88	25	2	-933.43	-6.06
3	7	-935.67	-6.36	13	7	-935.45	-5.88	25	3	-933.4	-6.07
4	0	-937.22	-6.58	14	0	-935.32	-6.53	25	4	-933.4	-6.07
4	1	-937.13	-6.57	14	1	-935.3	-6.53	25	5	-933.4	-6.07
4	2	-937.03	-6.56	14	2	-929.03	-5.86	25	6	-933.27	-6.05
4	3	-936.98	-6.55	14	3	-927.09	-6.39	25	7	-933.27	-6.05
4	4	-936.64	-6.55	14	4	-924.84	-5.78	26	0	-933.43	-6.55
4	5	-931.18	-6.18	14	5	-924.12	-5.82	26	1	-933.05	-6.58
4	6	-930.67	-6.16	14	6	-923.32	-5.51	26	2	-932.18	-6.6
4	7	-930.58	-6.14	14	7	-921.23	-5.87	26	3	-927.48	-5.95
5	0	-937.21	-6.24	15	0	-935.28	-6.58	26	4	-926.58	-6.16
5	1	-937.21	-6.24	15	1	-935.25	-6.58	26	5	-925.11	-6.07
5	2	-937.17	-6.24	15	2	-935.25	-6.58	27	0	-933.35	-5.79
5	3	-937.17	-6.24	15	3	-927.83	-6.07	27	1	-933.35	-5.79
5	4	-937.16	-6.24	15	4	-927.83	-6.07	27	2	-933.35	-5.79
5	5	-937.16	-6.24	15	5	-925.22	-5.87	27	3	-933.35	-5.79
5	6	-937.16	-6.24	15	6	-924.86	-5.81	27	4	-933.33	-5.79
5	7	-937.16	-6.24	15	7	-924.86	-5.81	27	5	-933.33	-5.79
5	8	-933.73	-6.05	16	0	-934.95	-5.79	27	6	-933.33	-5.79
5	9	-933.49	-6.05	16	1	-934.95	-5.79	27	7	-933.33	-5.79
5	10	-933.32	-6.05	16	2	-934.76	-5.75	28	0	-933.2	-6.58

6	0	-937.15	-6.73	16	3	-934.76	-5.75	28	1	-933.19	-6.58
6	1	-936.99	-6.72	16	4	-934.76	-5.75	28	2	-933.18	-6.59
6	2	-936.87	-6.71	16	5	-933.77	-5.67	28	3	-933.18	-6.57
6	3	-936.79	-6.71	16	6	-933.77	-5.67	28	4	-932.93	-6.66
6	4	-929.35	-6.25	16	7	-933.77	-5.67	28	5	-931.65	-6.83
6	5	-929.33	-6.26	17	0	-934.78	-6.49	28	6	-931.64	-6.82
6	6	-929.33	-6.26	17	1	-934.71	-6.51	29	0	-932.6	-6.47
6	7	-929.3	-6.27	17	2	-934.66	-6.5	29	1	-932.6	-6.47
7	0	-936.92	-6.14	17	3	-934.65	-6.49	29	2	-932.6	-6.47
7	1	-936.92	-6.14	17	4	-933.98	-6.38	29	3	-932.6	-6.47
7	2	-936.92	-6.14	17	5	-933.93	-6.42	29	4	-932.6	-6.47
7	3	-936.92	-6.14	17	6	-933.9	-6.42	29	5	-932.51	-6.46
7	4	-936.91	-6.14	17	7	-933.75	-6.38	29	6	-932.51	-6.46
7	5	-936.91	-6.14	18	0	-934.67	-6.4	29	7	-932.51	-6.46
7	6	-936.91	-6.14	18	1	-934.53	-6.39	29	8	-929.96	-6.03
7	7	-936.79	-6.13	18	2	-934.45	-6.39	29	9	-929.02	-6.03
8	0	-936.8	-6.66	18	3	-933.78	-6.29	30	0	-931.24	-6
8	1	-935.89	-6.54	18	4	-930.83	-6	31	0	-930.54	-6.16
8	2	-935.82	-6.54	18	5	-930.1	-5.93	31	1	-927.88	-5.94
8	3	-935.57	-6.52	18	6	-929.27	-6.03	31	2	-926.91	-6
8	4	-935.48	-6.54	18	7	-928.21	-5.85	32	0	-927	-6.09
8	5	-934.8	-6.45	19	0	-934.18	-6.05	33	0	-923.94	-5.95
8	6	-934	-6.38	19	1	-934.18	-6.05	33	1	-921.56	-7.07
8	7	-933.3	-6.4	19	2	-934.18	-6.05	33	2	-919.93	-6.32
9	0	-936.6	-6.52	19	3	-934.18	-6.05	33	3	-908.27	-5.03
9	1	-936.59	-6.54	19	4	-934	-6.06	33	4	-897.72	-4.85
9	2	-936.51	-6.51	19	5	-933	-6.11	33	5	-897.72	-4.84

9	3	-936.44	-6.51	19	6	-933	-6.11	34	0	-912.73	-5.73
9	4	-936.42	-6.53	19	7	-933	-6.11	34	0	-912.73	-5.73
9	5	-935.99	-6.46	20	0	-934	-6.38				
9	6	-935.8	-6.47	20	1	-933.99	-6.38				
9	7	-935.8	-6.47	20	2	-933.43	-6.4				
9	8	-935.8	-6.44	20	3	-933.38	-6.4				
9	9	-935.8	-6.44	20	4	-931.26	-6.21				
9	10	-934.7	-6.22	21	0	-933.94	-5.82				
9	11	-934.26	-6.16	21	1	-933.89	-5.81				
9	12	-933.28	-6.43	21	2	-933.89	-5.81				
9	13	-933.23	-6.47	21	3	-933.62	-5.8				
9	14	-933.18	-6.47	21	4	-933.62	-5.8				
